# Missed Antibiotic Doses, Microbiology Diagnostic Results, and Antibiotic Prescribing Patterns at Discharge at Two Paediatric Tertiary Hospitals in Zambia: Implications for Antimicrobial Stewardship

**DOI:** 10.3390/antibiotics15090838

**Published:** 2026-08-29

**Authors:** Chileshe Lukwesa-Musyani, Shadrick M. Ngosa, Mwelwa Chikombola, Davis Sondashi, Nayuda Kaonga, Evans Mwila Mpabalwani

**Affiliations:** 1 Institute of Basic Biological Sciences, Levy Mwanawasa Medical University, Lusaka P.O. Box 33991, Zambia; 2 School of Public Health and Social work, Texila American University, Plantation Providence, East Bank Demerara (EBD), Georgetown Lot 24-42, Guyana; 3 General Medicine, Arthur Davison Children’s Hospital, Ndola P.O. Box 240227, Zambia; shadrickmngosa@yahoo.com (S.M.N.); chikombolamwewa20@gmail.com (M.C.); 4 Pharmacy Department, Arthur Davison Children’s Hospital, Ndola P.O. Box 240227, Zambia; sondashidavies@yahoo.com; 5 Pharmacy Department, University Teaching Hospitals–Children’s Hospital, Lusaka P.O. Box 50110, Zambia; nayudakaonga@gmail.com; 6 Department of Paediatrics and Child Health, School of Medicine, University of Zambia, Lusaka P.O. Box 50110, Zambia; evansmwila@gmail.com

**Keywords:** antimicrobial stewardship, pediatric, missed doses, microbiological diagnostics, antibiotic prescribing at discharge

## Abstract

Background/objectives: Antimicrobial stewardship (AMS) is essential to optimize antibiotic use and limit antimicrobial resistance (AMR), particularly in pediatric populations where diagnostic uncertainty and system constraints complicate care. This study investigated critical AMS indicators, namely, missed antibiotic doses, microbiological diagnostics, and antibiotic prescribing at hospital discharge, which are key challenges that may affect treatment outcomes in hospitalized children under five years old. Methods: A prospective descriptive study was conducted in two tertiary pediatric hospitals in Zambia among children aged 29 days to 59 months. Information on missed antibiotic doses, microbiological investigations, and antibiotic prescribing at discharge from hospitalization was obtained from medical records and medication charts. Results: Patients experienced 1–8 missed doses, most commonly involving benzylpenicillin and ceftriaxone. Documentation of reasons for missed doses was largely absent. Microbiological testing was requested in 36.6% of patients, but only 14.8% of antibiotic prescriptions were supported by culture and susceptibility testing. Of requested tests, 80.9% of results were unavailable at discharge, limiting clinical utility. Only 19.1% of results were available, with a low culture positivity rate (27.3%). Overall, 41.8% of patients were discharged on antibiotics, predominantly from the WHO “Access” group (78%), though 22% were “Watch” antibiotics, and none from the “Reserve” group. Shorter hospital stay was significantly associated with discharge antibiotic prescribing (*p* = 0.0079; χ^2^ = 78.577, *p* < 0.001). Conclusions: Significant AMS gaps exist, including frequent missed doses, limited diagnostic support, and high discharge antibiotic use. Strengthening medication administration systems, improving laboratory capacity and turnaround times, and optimizing discharge prescribing are critical for enhancing pediatric AMS and reducing AMR.

## 1. Introduction

Antimicrobial stewardship (AMS) is a coordinated, evidence-based approach that promotes the rational, safe, and effective use of antimicrobial agents to optimize clinical outcomes while minimizing the emergence and spread of antimicrobial resistance (AMR). The growing global threat of AMR has been strongly linked to inappropriate and excessive antibiotic use, underscoring the importance of robust stewardship interventions across healthcare settings [1,2].

In pediatric populations, the implementation of AMS is particularly challenging due to clinical complexity, a high burden of infectious diseases, age-specific pharmacological considerations, and systemic constraints within healthcare systems [3,4,5,6]. This population is especially vulnerable to severe bacterial infections, including pneumonia, sepsis, and meningitis, where timely initiation and completion of appropriate antimicrobial therapy are critical to reducing morbidity and mortality [7,8,9,10]. The appropriate use of antibiotics in hospitalized children under five years of age therefore remains a foundation of high-quality clinical care and a key priority for AMS programs.

However, evidence indicates that suboptimal antibiotic practices occur across inpatient and transitional care settings. Two major areas of concern have been identified: (1) missed or omitted antibiotic doses during hospitalization, which may compromise therapeutic efficacy and contribute to treatment failure; (2) inappropriate, unnecessary, or prolonged antibiotic prescribing at the point of discharge. Studies have shown that a substantial proportion of pediatric discharge prescriptions are suboptimal in terms of antibiotic choice or duration, highlighting a critical gap in stewardship oversight. These studies have demonstrated that patients are frequently prescribed unnecessary, suboptimal, or prolonged courses of antibiotics at discharge, contributing to adverse drug events, increased healthcare costs, and the amplification of antimicrobial resistance (AMR) [11,12,13,14,15]. A USA study showed that in 30% of patients from children’s hospitals discharged on antibiotics, as high as 80% were suboptimal in terms of choice and days of therapy (DOT) [15]. Transitions of care such as discharge to home or transfer to another healthcare facility are recognized as high-risk periods for inappropriate continuation of antimicrobial therapy. Several drivers contribute to inappropriate antibiotic use at the point of discharge such as absence of culture and antimicrobial susceptibility testing (AST) results, prescriber uncertainty, lack of protocols for common infections, and limited AMS oversight as the focus is mostly on inpatient prescribing. Additionally, AMS opportunities are often missed in children with confirmed viral infections, where antibiotics are not discontinued despite evidence supporting a non-bacterial cause [16,17]. This reflects both diagnostic uncertainty and clinician caution, but also underscores the need for improved integration of laboratory diagnostics into clinical decision-making.

However, stewardship efforts are frequently complicated by diagnostic uncertainty, particularly in settings where laboratory capacity is limited. Delayed availability of laboratory results or, in some cases, the complete absence of microbiological and diagnostic data can significantly hinder timely, evidence-based clinical decision-making. This challenge is further amplified by the high prevalence of viral infections in young children, many of which present with clinical features that overlap with bacterial infections. Consequently, antibiotics are often initiated or continued empirically despite a viral etiology, contributing to unnecessary antimicrobial exposure and increased risk of AMR [11,12,18]. Point-of-care diagnostic testing using inflammatory biomarkers are increasingly used to support the diagnosis and management of infectious diseases and to improve antimicrobial stewardship. C-reactive protein (CRP) and procalcitonin (PCT) have demonstrated greater utility in differentiating bacterial from viral infections and in guiding decisions on antibiotic initiation and discontinuation. The World Health Organization recognizes access to appropriate diagnostic tests as a key component of diagnostic stewardship and antimicrobial stewardship programs, particularly in low- and middle-income countries where empirical antibiotic prescribing remains common due to limited laboratory capacity.

Antimicrobial stewardship guidance emphasizes regular review of antimicrobial therapy, optimization of treatment duration, and avoidance of unnecessary continuation of antibiotics, including at hospital discharge. These principles are consistent with the WHO AWaRe framework, which identifies fluoroquinolones and third-generation cephalosporins as Watch antibiotics and recommends their use be carefully monitored and restricted to situations in which they are clinically indicated [19]. A multinational consensus review stresses the need for optimal regimen, dose, duration, and route, supported by microbiology and local resistance patterns, and embedded within AMS programs that include audit and feedback [20].

Addressing the diagnostic, clinical, and operational challenges including delayed or unavailable laboratory results, missed inpatient doses, and suboptimal discharge prescribing is essential to improving patient outcomes and strengthening antimicrobial stewardship efforts in pediatric care. Against this backdrop, the present study aimed to (1) determine the frequency and underlying causes of missed antibiotic doses, (2) determine availability of laboratory results for decision-making, and (3) evaluate patterns of antibiotic prescribing at hospital discharge in a pediatric tertiary care setting. A comprehensive understanding of these factors is essential to inform targeted AMS interventions, improve medication safety, and promote the rational and effective use of antimicrobials in children.

## 2. Results

A total of 385 patient files were reviewed across the two participating tertiary pediatric hospitals: the University Teaching Hospitals—Children’s Hospital (UTHs-CH) in Lusaka Province with a bed capacity of about 365, and Arthur Davison Children’s Hospital (ADCH) in the Copperbelt Province with a bed capacity of 269. Of these, 179 (46.4%) records were obtained from UTHs-CH, while 206 (53.6%) were from ADCH.

Overall, male patients accounted for 56.9% (219/385) of the cohort with female patients accounting for 43.1% (166/385), with no statistically significant difference observed between the two hospitals (*p* = 0.58). Children under two years of age constituted the majority of the cohort, representing 239/385 (61.9%). Most of the patients admitted to the two hospitals, across all age groups, were prescribed antibiotics: 73.2% at UTHs-CH and 88.8% at ADCH.

### 2.1. Missed Antibiotic Doses

**Figure 1:**
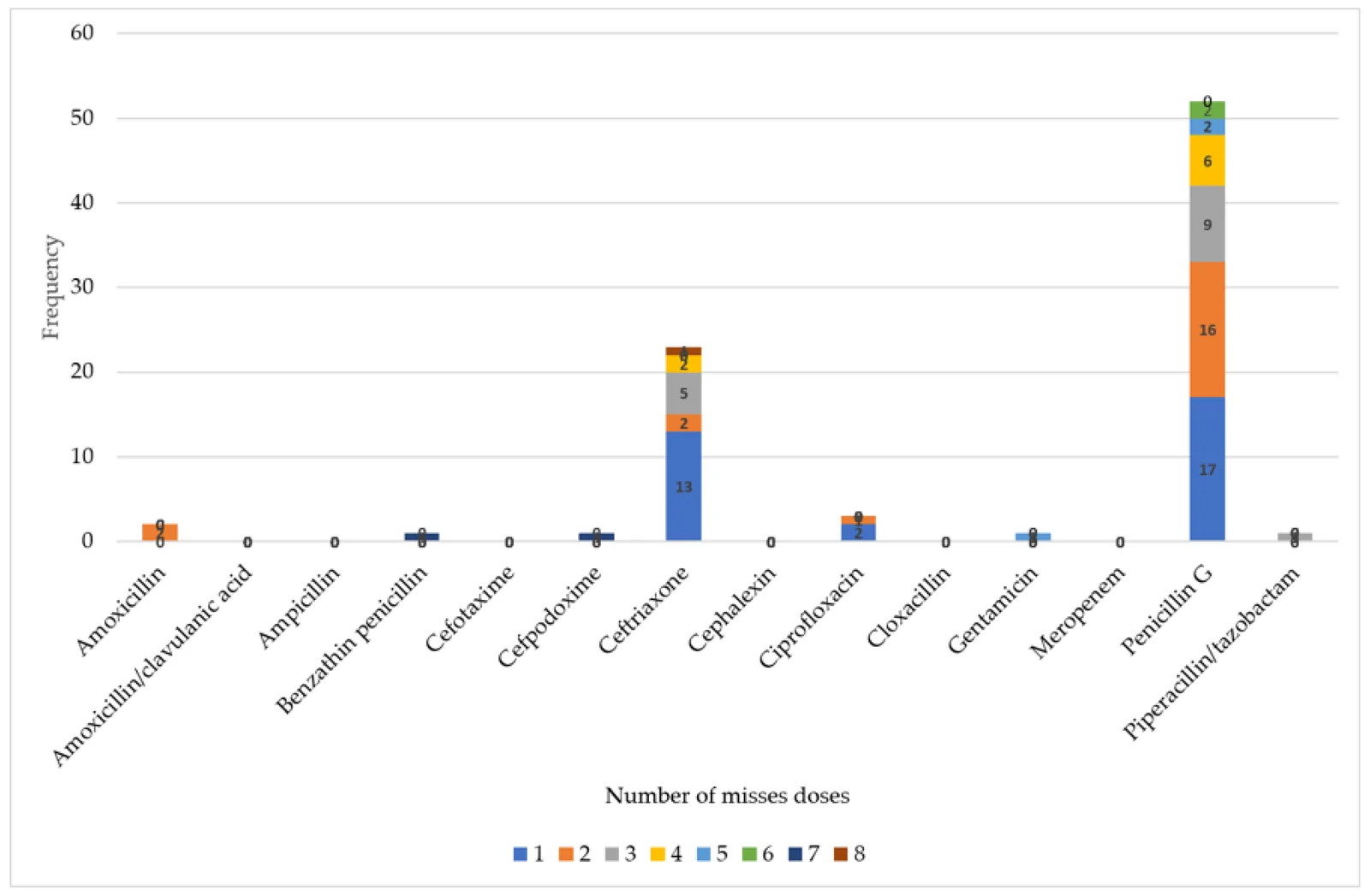
Frequency and number of missed antibiotic doses for different antibiotics.

The patients experienced between one and eight missed antibiotic doses during their course of treatment. The antibiotics most frequently prescribed were benzylpenicillin and ceftriaxone, accounting for 43% and 35% respectively. These were the mostly associated with missed doses. More than half of the affected patients missed one to two doses of benzylpenicillin across the entire treatment course, while approximately 20% missed at least one dose of ceftriaxone on separate days (Figure 1).

Documentation of the reasons for missed doses was notably limited. In almost all cases, no reason was recorded in the medication administration charts. Only one instance included a documented cause, identified as a “tissued cannula”, indicating intravenous access failure. There was no significant difference in the frequency of missed doses between the two hospitals (χ^2^ = 8.3256, *p*-0.4023; Table 1).

### 2.2. Microbiology Diagnostic Testing

Of the 385 sampled patient records, 314 documented antibiotic prescriptions and, among these, 36.6% (115/314) of the prescribed antibiotics had culture and antimicrobial susceptibility testing (AST) requested; the proportions were 14.8% (27/183) at ADCH and 67.2% (88/131) at UTHs-CH.

Despite these requests, the availability of microbiology results at the time of patient discharge was limited. Of the 115 cases with requested investigations, results were unavailable in 93/115 (80.9%) of cases. Among the 22/115 (19.1%) cases with available results, only 6/22 (27.3%) yielded positive findings, while the remainder were negative. At the facility level, results were unavailable at discharge in 87.5% of microbiology requests at UTHs-CH and 59.3% at ADCH (Table 2).

Out of the six laboratory tests that yielded the bacterial pathogens *Pseudomonas aeruginosa* and *Klebsiella pneumoniae* were isolated from an ear swab of a 6-month-old child with otitis media in severe acute malnutrition (SAM) who was treated with piperacillin/tazobactam. The laboratory results showed both organisms were susceptible to piperacillin/tazobactam. *Staphylococcus aureus* was from a blood culture of a 2-month-old female patient diagnosed with sepsis treated with a combination of penicillin and gentamicin. Cefoxitin was not tested to rule out methicillin-resistant *Staphylococcus aureus* (MRSA). *Enterobacter* species, *Enterococcus* species and *Proteus* species were from urine specimens.

Point-of-care (POC) tests were requested to support the management of patients admitted to the two pediatric hospitals. Laboratory investigations classified as “other” tests, rather than culture and antimicrobial susceptibility testing (AST), included full blood count (FBC), C-reactive protein (CRP), and erythrocyte sedimentation rate (ESR). FBC was the most frequently requested investigation, accounting for 88% of requests. “FBC only” (56%) and combinations of two or three POC investigations were also recorded, which included FBC and ESR (11%), FBC and CRP (3%), and FBC, CRP, and ESR (9%), while 12% of patients had no POC test requested. Although most FBC and some ESR results were available in the patients’ records, these data were not included in the present study. CRP, and procalcitonin testing services were unavailable during the study period; consequently, no results were recorded for these investigations. No requests for procalcitonin testing were documented.

As observed in this study, generally, there were no results available to guide escalation and de-escalation of antibiotics as observed in the scarcity of microbiology results. Therefore, frequency of escalation and de-escalation was generally not based on the availability of laboratory results. Out of 314 prescriptions, only 52 (16.5%) were modified, of which 23 (44.2%) were de-escalations, 19 (36.5%) were escalations, and 10 (19.2%) indicated no change as there was neither escalation nor de-escalation. Escalation was defined as a switch to or addition of an agent with a broader spectrum, additional coverage, or a change from oral to intravenous therapy. Conversely, de-escalation of therapy was characterized by a switch from intravenous to oral administration, or a change to an agent with less broad-spectrum coverage. The therapy was classified as unchanged when neither escalation nor de-escalation was noted.

### 2.3. Antibiotic Prescribing at Discharge from Hospital

Among the 385 patients included in this study, 161 (41.8%) were discharged with antibiotic therapy. Patients who received antibiotics during hospitalization were significantly more likely to be prescribed antibiotics at discharge compared with those who did not receive inpatient antibiotic therapy. Of the 314 patients who received antibiotics during hospitalization, 146 (46.5%) were discharged on antibiotics, compared with 168 (53.5%) who were not. Among the 71 of the 385 patients who were not prescribed antibiotics during hospitalization, 15 (21.1%) were nevertheless prescribed antibiotics at hospital discharge, and conversely 56 (78.9%) were not prescribed antibiotics at discharge.

Receiving antibiotics during hospitalization was significantly associated with antibiotic prescribing at discharge (OR = 3.24; χ^2^ ≈ 15.3, df = 1; *p* < 0.001), indicating that patients who received inpatient antibiotics had approximately three times higher odds of being discharged on antibiotics than those who did not receive inpatient antibiotics. However, the clinical rationale for discharge antibiotic prescriptions was not documented, limiting assessment of their appropriateness. Furthermore, other potentially relevant determinants of discharge prescribing, including age, diagnosis, illness severity, microbiological results, and length of hospital stay, were not assessed and may have contributed to the observed association.

The most commonly prescribed antibiotics at discharge were: amoxicillin (29%), amoxicillin/clavulanic acid (16%), phenoxymethylpenicillin (11%), cephalexin (10%), ciprofloxacin (9%), and cefixime (6%). Other antibiotics included azithromycin, cefpodoxime, benzathine penicillin, cefuroxime, cloxacillin, phenoxy penicillin, ampicillin/cloxacillin, ampicillin, trimethoprim/sulfamethoxazole, nitrofurantoin and metronidazole (Table 3).

According to the pharmacological classification, classification of discharge antibiotics according to the WHO AWaRe framework showed that the majority (78%) belonged to the Access group, while 22% were classified as Watch antibiotics. No Reserve antibiotics were prescribed. The variance in antibiotic distribution across AWaRe categories was 28.5.

**Figure 2:**
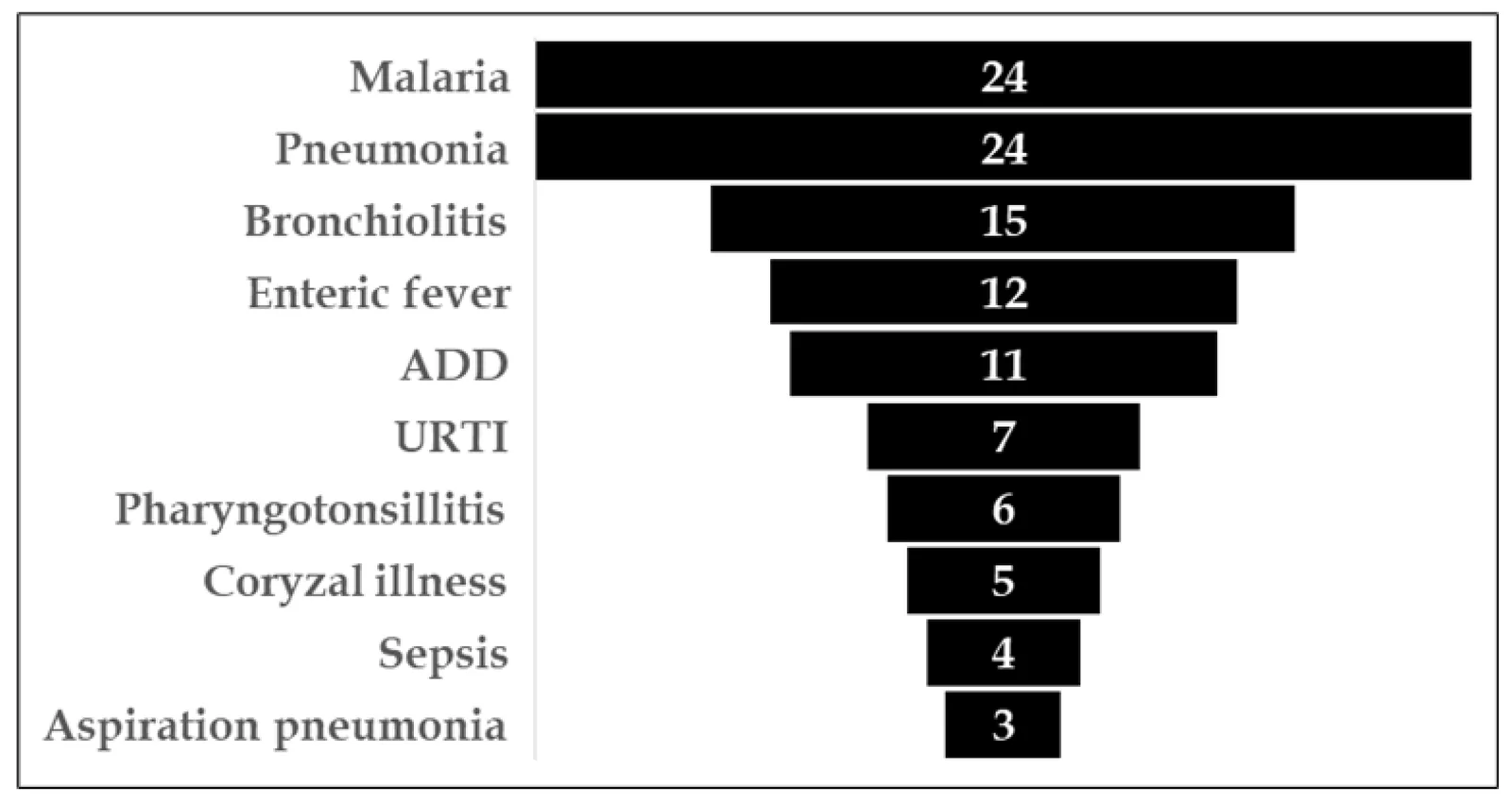
Top 10 indications in which antibiotics were prescribed at discharge from hospitalization (ADD—Acute diarrheal disease, URTI—Upper respiratory disease).

The top ten diagnoses in which antibiotics were prescribed post hospitalization are illustrated in Figure 2. Predominant diagnoses included malaria, pneumonia, and respiratory illnesses, primarily of known viral origin based on clinical diagnosis, such as bronchiolitis, coryzal illness, and other upper respiratory infections. Prescribing with a diagnosis of malaria during hospitalization had no documentation on clinical rationale, and this study did not include clinician interviews.

### 2.4. Association Between Length of Antibiotic Therapy During Hospitalization and Discharge on Antibiotics

The duration of inpatient antibiotic therapy ranged from 1 to 18 days, with a mean duration of 9.5 days (SD ± 5.3), a median of 9.5 days, and an interquartile range (IQR) of 9 days, reflecting moderate variability in treatment duration. A binary logistic regression analysis was performed to assess the association between duration of inpatient antibiotic therapy (days) and the likelihood of being discharged on antibiotics. The dependent variable was discharge on antibiotics (Yes/No), while duration of therapy (days) was included as the independent variable. The fitted logistic regression model showed an overall negative relationship between duration of therapy and the probability of discharge on antibiotics.

The odds ratio (OR) for duration of therapy was 0.88 per additional day of treatment, indicating that each additional day of inpatient antibiotic therapy was associated with an approximately 12% reduction in the odds of being discharged on antibiotics. The fitted probability curve demonstrated that the likelihood of discharge on antibiotics generally declined with increasing duration of therapy, although there was variability in the observed proportions at individual durations because of the relatively small sample sizes at longer treatment durations.

**Figure 3:**
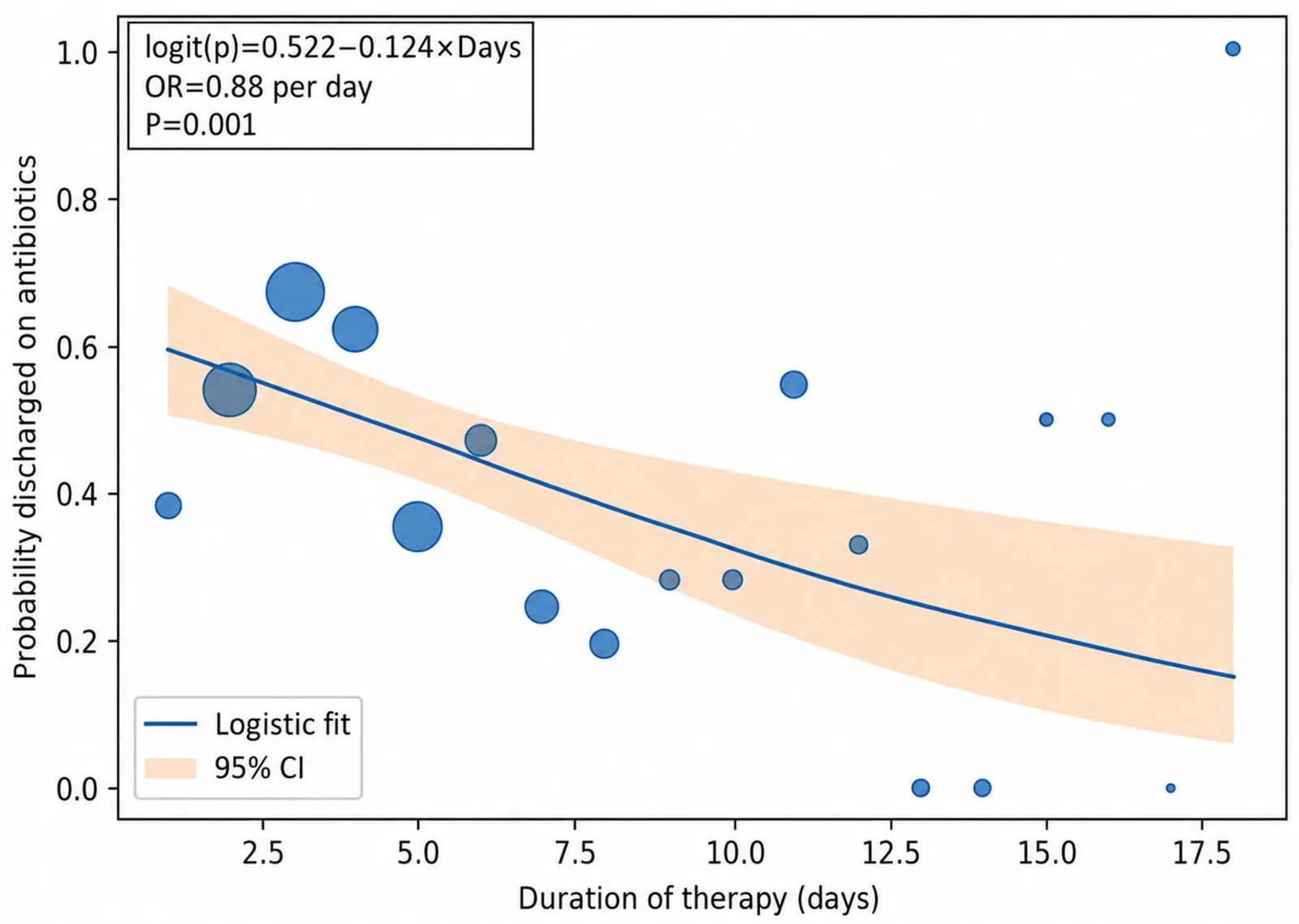
Association between probability of discharge on antibiotics post hospitalization and Duration of antibiotic therapy.

The observed proportions showed that the highest probability of discharge on antibiotics occurred among children receiving 2–4 days of therapy, after which the probability generally decreased as treatment duration increased. The wider confidence intervals at durations beyond 10 days reflected the limited number of patients in these categories (Figure 3).

## 3. Discussion

This study identified two critical gaps in AMS within pediatric inpatient care: (1) frequent missed antibiotic doses during the duration of antibiotic therapy; (2) significant rates of antibiotic prescribing at hospital discharge. Both findings highlight systemic challenges that have important implications for clinical outcomes and AMR.

Missed antibiotic doses were most commonly associated with benzylpenicillin, a β-lactam antibiotic that typically requires multiple daily administrations. This pattern likely reflects operational constraints such as high nursing workload, competing clinical priorities, and challenges in maintaining strict dosing schedules. Given that β-lactam antibiotics exhibit time-dependent pharmacodynamics, maintaining serum drug concentrations above the minimum inhibitory concentration is essential for therapeutic efficacy. Consequently, missed doses may lead to suboptimal treatment, increased risk of clinical failure, prolonged hospitalization, and the emergence of resistant organisms [21].

The finding that approximately 20% of ceftriaxone doses were missed despite its convenient once-daily dosing suggests that medication omissions are not solely related to dosing frequency but may reflect broader systemic issues in medication administration processes. These may include workflow inefficiencies, documentation gaps, and limited accountability mechanisms. Similar findings from previous studies conducted at UTHs-CH indicate that missed doses are a persistent problem in this setting, underscoring the need for system-level interventions such as improved workflow design, routine adherence monitoring, and targeted staff education on the clinical importance of timely antibiotic administration [22,23].

There are identified substantial gaps in the utilization and clinical impact of microbiology diagnostic testing among pediatric inpatients receiving antibiotics. Only 36.6% of patient records with antibiotic prescriptions had a documented request for microbiological investigations (culture and AST). When analyzed per antibiotic prescription, this proportion was even lower (14.8%), indicating that the majority of antimicrobial therapy was initiated empirically without microbiological confirmation and results were not available to support escalation and de-escalation of antibiotic therapy. The low testing rates and delayed or unavailable laboratory results suggests that both diagnostic and AMS systems require strengthening. Despite non-availability of laboratory results, there was de-escalation and escalation of antibiotic therapy. According to a study in early onset neonatal sepsis by Nazedah et al., empiric antibiotic de-escalation was encouraged in early onset neonatal sepsis as it was observed that there were comparable treatment outcomes to those in neonates without antibiotic de-escalation [24].

While UTHs-CH had higher test request rates, the lack of timely results at both institutions underscores that test utilization alone is insufficient without concurrent improvements in laboratory efficiency and result-reporting systems. It is therefore important to strengthen diagnostic stewardship practices, including protocols for timely specimen collection prior to antibiotic initiation, improve both yield and clinical relevance of results. These findings are consistent with reports from other low- and middle-income countries (LMICs), where diagnostic stewardship remains suboptimal. In a multicenter pediatric study in South Africa only 28% of patients had cultures requested, with results available in just 38% of cases [23]. Similarly, point prevalence surveys conducted across African and Asian settings have demonstrated low rates of microbiological testing prior to antibiotic initiation, often below 40%, reflecting reliance on empiric therapy due to systemic and operational constraints [25,26,27]. High-income regions report substantially greater use of microbiological diagnostics. European studies show cultures are obtained for most severe or hospitalized pediatric infections, while global data indicate variable prevalence of targeted antibiotic therapy based on microbiological findings; a worldwide prevalence study of pediatric healthcare-associated infections reported antibiotic prescribing rates ranging from 23.8% in Africa to 39.4% in Australia [28]. These differences likely reflect disparities in diagnostic capacity and antimicrobial stewardship practices. Developed countries are supported by well-resourced laboratory infrastructure and integrated AMS stewardship programs. These differences highlight persistent inequities in access to diagnostic services and their integration into clinical care.

The low availability of results at discharge is a critical finding and reflects several well-documented challenges in low- and middle-income country (LMIC) settings. These include prolonged laboratory turnaround times, limited laboratory capacity, shortages of trained personnel, stock-outs of culture media and reagents, and inefficient specimen transport systems [29,30]. It is worth investing in modern diagnostic technologies, such as matrix-assisted laser desorption ionization/time of flight (MALDI-TOF) for organism identification, and multiplex polymerase chain reaction (PCR) to allow for rapid and accurate identification of infectious pathogens and certain AMR genes, which reduces the turnaround time of laboratory results [31,32]. Even among the limited number of available results only 27.3% were culture-positive. Strong diagnostic stewardship practices, including protocols for timely specimen collection prior to antibiotic initiation, improve both yield and clinical relevance of results. However, it is worth noting that this study evaluated the availability of microbiology results to support antimicrobial stewardship, rather than the appropriateness of requesting cultures for every patient. Not all antibiotic prescriptions in hospitalized patients may necessarily require microbiological culture and antimicrobial susceptibility testing (AST). The decision to obtain microbiological specimens is guided by the clinical syndrome, severity of illness, suspected pathogen, likelihood of bacterial infection, and the potential for test results to influence antimicrobial management.

The findings indicate that routine laboratory support for the management of pediatric patients in the two hospitals relied predominantly on the full blood count (FBC), which accounted for 88% of all requested point-of-care and inflammatory marker investigations. This reliance is not unexpected in resource-limited settings, where FBC is widely available, relatively inexpensive, and provides rapid information on leukocyte counts and other hematological parameters that may suggest infection. However, FBC is a non-specific marker of inflammation and cannot reliably distinguish bacterial from viral infections. Consequently, although it may support the overall clinical assessment, its value for guiding antimicrobial prescribing is limited when used in isolation. Similarly, erythrocyte sedimentation rate (ESR), which was requested either alone or in combination with FBC, is a non-specific indicator of inflammation that has limited utility in the diagnosis of acute bacterial infections because of its slow response to changes in disease activity.

An important finding was the absence of C-reactive protein (CRP) and procalcitonin testing during the study period. Although CRP was requested for a proportion of patients, the service was unavailable and no results were recorded, while no requests for procalcitonin testing were documented because the assay was not available in either hospital. These findings reflect important gaps in diagnostic capacity that may limit clinicians’ ability to differentiate bacterial from viral infections and to support evidence-based antimicrobial prescribing. CRP and procalcitonin have been shown to improve diagnostic accuracy when interpreted alongside clinical findings, with procalcitonin demonstrating particular value in antimicrobial stewardship programs by supporting decisions to initiate, withhold, or discontinue antibiotic therapy [33]. The absence of CRP and procalcitonin testing may therefore contribute to greater reliance on empirical treatment, especially that culture and antimicrobial susceptibility test results were also delayed or unavailable. Strengthening access to appropriate inflammatory biomarkers, together with timely microbiological diagnostics, would enhance diagnostic stewardship by enabling more targeted antibiotic prescribing, reducing unnecessary antimicrobial exposure, and improving the quality of care for hospitalized children. Overall, these findings are consistent with regional and global evidence demonstrating that limited access to timely and reliable microbiological diagnostics remains a major barrier to optimal antimicrobial use in pediatric care [29].

The high proportion of patients (41.8%) discharged on antibiotics represents an additional and significant stewardship concern. The observed association between shorter hospital stays and increased likelihood of discharge antibiotic prescribing may suggest a tendency toward precautionary continuation of therapy beyond inpatient care, rather than decisions guided strictly by clinical indications. This finding is consistent with global evidence identifying transitions of care as high-risk periods for inappropriate antimicrobial use, often resulting in unnecessary or prolonged antibiotic courses [12,14].

The observation that patients who received antibiotics during hospitalization were significantly more likely to be discharged on antibiotics than those who did not receive inpatient antibiotics is clinically plausible. Patients requiring antibiotics during hospitalization are likely to have active bacterial infections that may not have fully resolved by the time they are clinically stable for discharge. Consequently, clinicians often prescribe oral antibiotics to complete the recommended treatment course in the outpatient setting. This practice supports continuity of care while avoiding unnecessarily prolonged hospitalization.

Although most discharge prescriptions were from the Access category of the WHO AWaRe classification, the use of Watch antibiotics including fluoroquinolones and third-generation cephalosporins is concerning [12,13,34]. This pattern, together with suboptimal choices and durations, undermines prudent antibiotic stewardship. These agents are associated with a higher propensity to drive AMR and are recommended for more restricted use in stewardship frameworks. Pediatric AMS guidelines emphasize limiting the routine use of such antibiotics and ensuring that their prescription is supported by clear clinical justification. Different interventions have been proposed, such as adherence to institutional treatment guidelines, and trainings [35,36].

Missed doses reflect the quality and safety of medication administration, while discharge prescribing reflects the quality of prescribing decisions and influences antibiotic use after patients leave the hospital. Both practices are important quality indicators within antimicrobial stewardship programs. Therefore, together, they affect treatment outcomes, patient safety, healthcare resource utilization, and the development and spread of antimicrobial resistance. Consequently, monitoring both indicators provides valuable information for improving healthcare quality and strengthening antimicrobial stewardship interventions.

Addressing all these challenges requires a multifaceted approach. Key interventions include the implementation of structured antibiotic review processes at discharge, development and enforcement of evidence-based guidelines for duration of therapy in common pediatric infections, and the integration of pharmacist-led stewardship activities. Additionally, systems should be established to monitor and reduce missed doses, alongside strengthening documentation practices to ensure that reasons for omissions are consistently recorded. Collectively, these strategies have the potential to reduce unnecessary antibiotic exposure, optimize treatment outcomes, and mitigate the risk of AMR.

## 4. Materials and Methods

This prospective descriptive study was conducted over a three-month period at two tertiary-level pediatric referral hospitals located in the Copperbelt and Lusaka provinces of Zambia. Data were collected through a systematic review of inpatient medical records and medication administration charts to comprehensively capture clinical characteristics, antimicrobial prescribing practices, and treatment-related information.

A total of 385 files of pediatric patients aged 29 days to 59 months (under five years old excluding neonates) who were admitted to either of the two participating hospitals during the study period were sampled. All eligible patients within this age group were included regardless of whether they received antibiotic therapy. This approach enabled a comprehensive assessment of antimicrobial prescribing practices and diagnostic utilization among hospitalized children.

Data were collected using a structured tool adapted from the World Health Organization (WHO) Point Prevalence Survey (PPS) methodology version 1.1 Geneva: World Health Organization; 2018, to ensure standardized data collection and facilitate comparability across the two study sites. This tool designed for PPS was modified to include collection of prospective data such as length of hospital stay, length of antibiotic therapy, follow-up of availability of laboratory results (culture and antibiotic susceptibility testing (AST)) and prescribed antibiotics at point of discharge from hospital. This also enabled comprehensive collection of data on frequency and reasons for missed antibiotic doses following complete information on drug administration during hospital stay.

The patient drug charts were inspected for missed drug administration across the entire treatment course to identify any missed antibiotic doses, from the initiation of prescribed therapy until completion of the treatment course or at hospital discharge, whichever occurred first. A missed antibiotic dose was one that was ordered but never given (a completely missed dose) or a dose that was not given within an hour before or after the planned time (an off-schedule missed dose). Where a drug typically was administered multiple times daily, this referred to missed doses across the entire treatment course, and in the case of once daily dosing, this referred to omission on two separate days. Healthcare practitioners sign the chart when a drug is administered, and indicate with a dash when not administered. The number of dashes were counted as missed doses. Patients with no missed doses were assigned a value of zero. Medication administration records were also reviewed to identify and classify the reasons for missed doses using predefined categories: medication stock shortages, caregiver inability to procure medication, patient-related factors (e.g., the patient was asleep), and clinical or technical factors (e.g., difficulty obtaining intravenous access). If there were multiple contributing factors, this was to be categorized as “multiple reasons” and where no documented reason was available, it was categorized as “unknown”. Patient records were also reviewed to determine whether culture and AST results were available during hospitalization and/or at the point of discharge from hospital.

Conventional methods were used for culture and identification of microorganisms during this study. while Antimicrobial susceptibility testing was by the Kirby–Bauer disc diffusion method. The AST, and quality control methods were conducted according to the Clinical and Laboratory Standard Institute (CLSI) guidelines. The files were reviewed at discharge to follow up on laboratory results.

Information was collected to determine whether antibiotics were prescribed at hospital discharge from hospitalization. For patients discharged on antibiotics, antimicrobial agents prescribed, their pharmacological class, and their classification according to the WHO Access, Watch, and Reserve (AWaRe) framework were determined to assess prescribing patterns and alignment with antimicrobial stewardship principles. Records were reviewed to find out in which patient diagnoses antibiotics were prescribed. The antibiotics prescribed at hospital discharge, and the associated patients’ clinical diagnosis during admission were recorded.

Data were analyzed using Microsoft Excel and IBM SPSS Statistics version 27.0.1 for Windows, Version 27. Descriptive statistics were used to summarize the data. Categorical variables were presented as frequencies and percentages. Associations between categorical variables, including comparisons between hospitals, were assessed using Pearson’s chi-square (χ^2^) test. Binary logistic regression analysis was performed to examine associations between length of antibiotic therapy and antibiotic prescribing at discharge from hospital. Statistical significance was set at *p* < 0.05 for all analyses.

## 5. Conclusions

Missed antibiotic doses and high rates of discharge antibiotic prescribing represent significant challenges to effective AMS in pediatric healthcare settings. These issues reflect both operational and clinical decision-making gaps that can adversely affect patient outcomes and contribute to the growing burden of antimicrobial resistance.

Targeted interventions are required to improve the reliability of medication administration and to ensure that antibiotics prescribed at discharge are clinically indicated, appropriately selected, and optimally dosed. Strengthening stewardship efforts at both the point of care and during transitions of care is essential to promote rational antimicrobial use, improve patient safety, and reduce the risk of resistance in pediatric populations. There is an urgent need to strengthen laboratory infrastructure and human resource capability to effectively enhance AMS.

## Figures and Tables

**Table 1:** Frequency of number of missed antibiotic doses for the two hospitals.

No. of Missed Doses	ADCH		UTHs-CH	
	Total	%	Total	%
0	126	75.4	79	64.8
1	16	9.6	16	13.1
2	11	6.6	10	8.2
3	9	5.4	6	4.9
4	3	1.8	5	4.1
5	1	0.6	2	1.6
6	1	0.6	1	0.8
7	0	0.0	2	1.6
8	0	0.0	1	0.8
	167	100.00	122	100

**Table 2:** Culture and antibiotic susceptibility test results availability at discharge from hospitalization at the two hospitals.

Hospital	Negative Culture Result	Positive Culture/AST Result	No Culture/AST Results Available at Discharge	No Culture/AST Test Requested	Total
ADCH	37.0% (10)	3.7% (1)	59.3% (16)	179	206
UTHs-CH	6.8% (6)	5.7% (5)	87.5% (77)	91	179
All	13.9% (16)	5.2% (6)	80.9% (93)	270	385

**Table 3:** Antibiotics, frequency and AWaRe classification of antibiotics prescribed at discharge from hospitalization.

Antibiotic	% (No.)	Classification	WHO AWaRe
Amoxicillin	29 (47)	Penicillins	Access
Amoxicillin/clavulanic acid	16 (26)	Beta-lactam/beta-lactamase inhibitor	Access
Phenoxy methyl penicillin	11 (18)	Penicillins	Access
Cefalexin	10 (16)	1st-generation cephalosporin	Access
Ciprofloxacin	9 (14)	Fluoroquinolone	Watch
Cefixime	6 (10)	3rd-generation cephalosporin	Watch
Azithromycin	4 (6)	Macrolides	Watch
Benzathine penicillin	2 (3)	Penicillins	Access
Cloxacillin	2 (4)	Penicillins	Access
Cefpodoxime	2 (4)	3rd-generation cephalosporin	Watch
Sulfamethoxazole/ trimethoprim	2 (3)	Sulfonamide trimethoprim combination	Access
Cefuroxime	1 (1)	2nd-generation cephalosporin	Watch
Ampicillin	1 (1)	Penicillins	Access
Ampicloxacillin	1 (1)	Not recommended	N/A
Amoxicillin and Metronidazole	1 (1)	Penicillins/imidazole	Access/Access
Cloxacillin and Metronidazole	1 (1)	Penicillins/imidazole	Access/Access
Metronidazole oral	1 (1)	Imidazole	Watch
Nitrofurantoin	1 (1)	Nitrofuran derivatives	Access
Other	1 (1)	N/A	N/A

## Data Availability

The antibiotic data utilization form was converted to a Google form format and an Excel format dataset was generated. Qualitative data on AMS were collected using a checklist. Data sharing is restricted by the conditions of ethical approval and institution policies. De-identified aggregated data are presented in the manuscript and individual level data can only be accessed with prior approval from the ethics committee and hospital authorities.

## Abbreviations

The following abbreviations are used in this manuscript:

ADCH	Arthur Davison Children’s Hospital
AMS	Antimicrobial Stewardship
AMR	Antimicrobial Resistance
AST	Antibiotic Susceptibility Testing
AWaRe	Access, Watch, Reserve
CLSI	Clinical Laboratory Standards Institute
CRP	C-Reactive Protein
DOT	Days of Therapy
ESR	Erythrocyte Sedimentation Rate
FBC	Full Blood Count
LMICs	Low- and Middle-Income Countries
MALDI-TOF	Matrix-Assisted Laser Desorption Ionization/Time of Flight
PCR	Polymerase Chain Reaction
PPS	Point Prevalence Survey
UTHs-CH	University Teaching Hospitals—Children’s Hospital
WHO	World Health Organization
